# Preoperative prediction of recurrence risk factors in operable cervical cancer based on clinical-radiomics features

**DOI:** 10.3389/fonc.2025.1492494

**Published:** 2025-02-28

**Authors:** Xue Du, Chunbao Chen, Lu Yang, Yu Cui, Min Li

**Affiliations:** ^1^ Department of Oncology, Affiliated Hospital of North Sichuan Medical College, Nanchong, China; ^2^ Department of Clinical Medicine, North Sichuan Medical College, Nanchong, China

**Keywords:** cervical cancer, radiomics, recurrence risk stratification, machine learning, predictive model

## Abstract

**Objective:**

To investigate the value of preoperative prediction of risk factors for recurrence of operable cervical cancer based on the radiomics features of biparametric magnetic resonance imaging (bp-MRI) combined with clinical features.

**Method:**

A retrospective collection of cervical cancer cases undergoing radical hysterectomy + pelvic and/or para-aortic lymph node dissection at the Affiliated Hospital of North Sichuan Medical College was conducted. Region of interest (ROI) was outlined using the 3D Slicer software, and radiomics after feature extraction and feature screening was performed using the least absolute shrinkage and selection operator (LASSO) algorithm. Logistic regression algorithms were used to construct a fusion clinical-radiomics model to visualize nomograms. Receiver operating characteristic (ROC), DeLong test, calibration curve (CC), and decision curve (DC) were used to evaluate the predictive performance and clinical benefit of the model.

**Result:**

A total of 99 patients with cervical cancer were included in this study, with 79 and 20 cases in the training and test groups, respectively. Seventeen key features were selected for radiomics model construction. Three clinical features were screened to construct a clinical model. A fusion model of the radiomics model combined with the clinical model was constructed. The area under the curve (AUC) values in the training group were 0.710 (95% CI 0.602–0.819), 0.892 (95% CI 0.826–0.958), and 0.906 (95% CI 0.842–0.970), for the comparative clinical model, radiomics model, and fusion model, respectively, and the AUC values in the testing group were 0.620 (95% CI 0.366–0.874), 0.860 (95% CI 0.677–1.000), and 0.880 (95% CI 0.690–1.000), respectively. The DeLong test showed a statistically significant difference between the AUC values of the fusion model and the clinical model (*p* < 0.05). Decision curve analysis (DCA) showed that the fusion model had the greatest net benefit when the threshold probability was approximately 0.5.

**Conclusion:**

The fusion model constructed based on bp-MRI radiomics features combined with clinical features provides an important reference for predicting the risk status of recurrence in operable cervical cancer. The findings of this study are preliminary exploratory results, and further large-scale, multicenter studies are needed to validate these findings.

## Introduction

According to global cancer statistics, the incidence of cervical cancer ranks 14th among all cancers and is the fourth most common cancer among women worldwide, seriously affecting women’s physical and mental health (1). Treatment modalities and prognosis of cervical cancer depend largely on the stage of the primary diagnosis, and treatment options for early-stage and locally invasive cervical cancer include simple hysterectomy or radical hysterectomy plus pelvic and/or para-abdominal aortic lymph node dissection (2). The 5-year survival rate of cervical cancer treated with surgery can reach 80%–90%, but 20%–30% of patients still develop recurrence or metastasis within 3 years (3). In patients with poor prognostic factors after radical hysterectomy, the addition of postoperative adjuvant therapy is recommended to reduce the risk of recurrence and improve progression-free survival. The International Federation of Gynecology and Obstetrics (FIGO) 2021 guidelines for cervical cancer state that radical radiotherapy or concurrent chemoradiotherapy (CCRT) is preferred for patients who may require postoperative radiotherapy in order to avoid treatment-related complications (4). Establishing a risk prediction model before treatment and accurately assessing risk factors for recurrence and risk stratification of cervical cancer patients can help guide the clinic, promote the development of individualized treatment strategies, reduce toxic side effects and economic pressure caused by multimodal treatment, and improve the quality of survival.

In 2010, Gillies RJ et al. (5) first proposed the concept of radiomics. In 2012, Lambin P et al. (6) further refined the concept of radiomics, defined as the high-throughput extraction of a large number of quantitative features from imaging images and the transformation of the data in medical images into a mineable data space with high resolution by automated or semiautomated analytical methods, allowing for comprehensive, non-invasive, and quantitative observation of spatial and temporal heterogeneity of tumors. Wu Q et al. (7), based on sagittal T2-weighted imaging (T2WI) and axial apparent diffusion coefficient (ADC) images, extracted radiomics features from intra-tumor and peri-tumor tissues, and established a support vector machine (SVM) model to predict the lymph node status of cervical cancer; the area under the curve (AUC) values and sensitivity were 0.895 and 94.3% and 0.847 and 100% in the training and testing groups, respectively, and the model had promising performance in predicting cervical cancer lymph node metastasis. Wang T et al. (8) developed an SVM model based on the radiomics features of T2WI and T2WI combined with diffusion-weighted imaging (DWI), which showed favorable performance in the assessment of paracervical infiltration and can be used as a complementary tool to provide individualized treatment options for patients with early-stage cervical cancer. Du W et al. (9) extracted radiomics features from T2WI to establish an SVM model for preoperative non-invasive prediction of lymphovascular space invasion (LVSI) status in cervical cancer, and the results showed an AUC of 0.925 with an accuracy of 87.5% in the training group and an AUC of 0.911 with an accuracy of 84.0% in the testing group. Radiomics has shown high diagnostic efficacy in the prediction of early-stage cervical cancer risk factors and has good potential for clinical application. However, the current studies mainly focus on the construction of prediction models for single risk factors. Benedetti-Panici et al. (10) found that pelvic lymph node metastasis was always associated with parietal uterine infiltration, revealing that individual risk factors are not independent but interrelated. Therefore, predictive models for a single risk factor alone cannot comprehensively assess the recurrence risk status of cervical cancer.

In this study, based on preoperative biparametric magnetic resonance imaging (bp-MRI) images, high-throughput extraction of quantitative features combined with clinical data through a radiomics approach was used to construct an individualized prediction model for the stratification of risk factors for the recurrence of operable cervical cancer in order to comprehensively and accurately assess the risk of recurrence, achieve preoperative accurate stratification of the risk of recurrence, and assist in the clinical development of an individualized treatment strategy.

## Materials and methods

### Characteristics of patients

**Figure 1:**
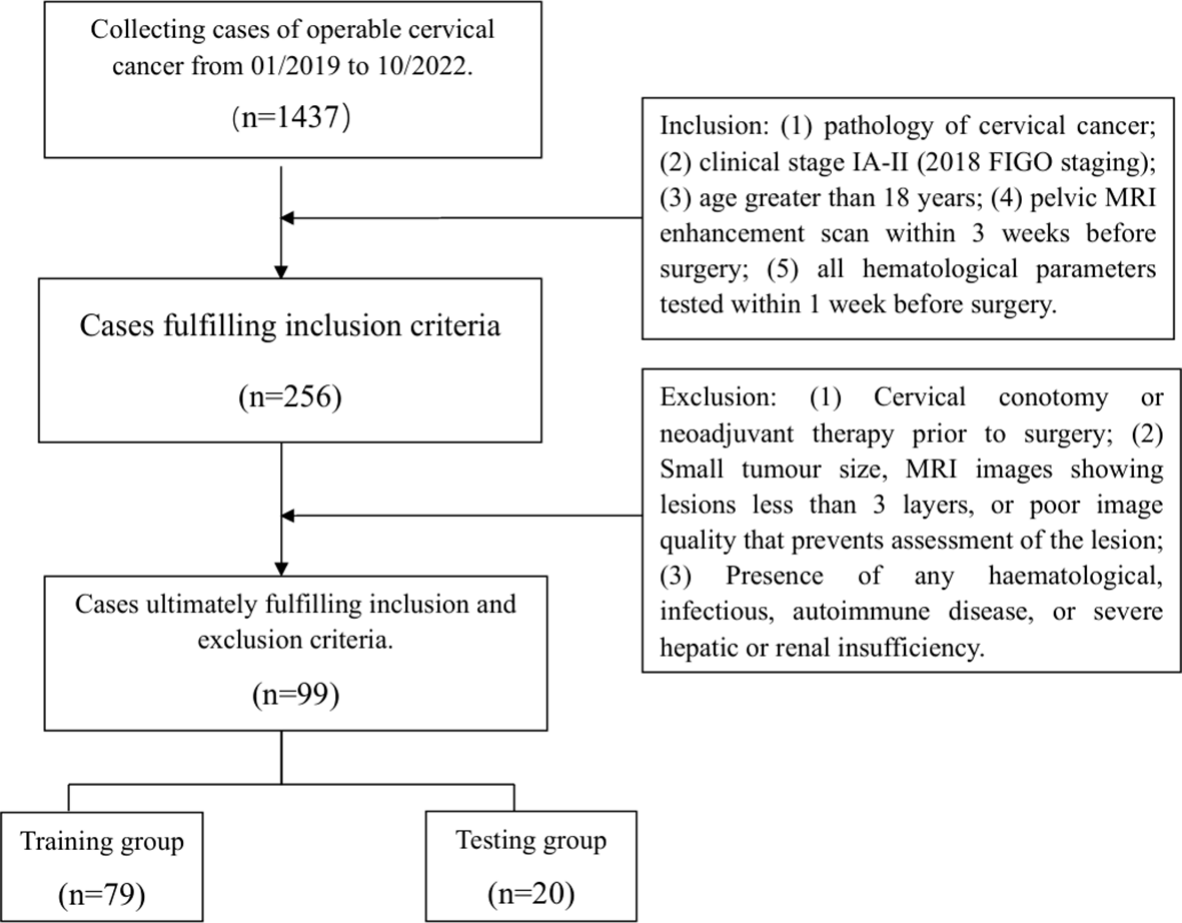
The flowchart of case screening.

A total of 99 cases of cervical cancer undergoing radical hysterectomy + pelvic and/or para-abdominal aortic lymph node dissection at the Affiliated Hospital of North Sichuan Medical College were retrospectively collected and randomly divided into a training group and a testing group in a ratio of 8:2 for the construction and validation of the prediction model. **Figure 1** shows the flowchart of case screening.

### Clinical laboratory and pathology data

Clinical data included age, menstrual status, number of pregnancies, deliveries and abortions, and clinical stage. Laboratory parameters included neutrophil count, lymphocyte count, monocyte count, platelet count, hemoglobin (Hb), red cell distribution width (RDW), plasma fibrinogen, albumin, squamous cell carcinoma antigen (SCCA), lymphocyte-to-monocyte ratio (LMR), neutrophil-to-lymphocyte ratio (NLR), platelet-to-lymphocyte ratio (PLR), systemic inflammatory response index (SIRI), systemic immunoinflammatory index (SII), albumin-to-fibrinogen ratio (FAR), and prognostic nutritional index (PNI). Pathological data included histological type, metastatic lymph nodes, para-uterine infiltration, surgical margins, LVSI, depth of interstitial infiltration (DSI), and tumor size (TS).

### Risk stratification

High-risk factors included lymph node metastasis (+), para-uterine infiltration (+), and surgical margins (+) (11); fulfilling any one of them was classified as a high-risk group. Intermediate-risk factors included LVSI (+), DSI > 1/3, and TS ≥ 4 cm; satisfying any two were classified as intermediate-risk group (12). Further, the high-risk and intermediate-risk groups were labeled the positive-risk (PR) group, while the other case groups were recorded as the low-risk (LR) group.

### Image collection and segmentation

**Figure 2:**
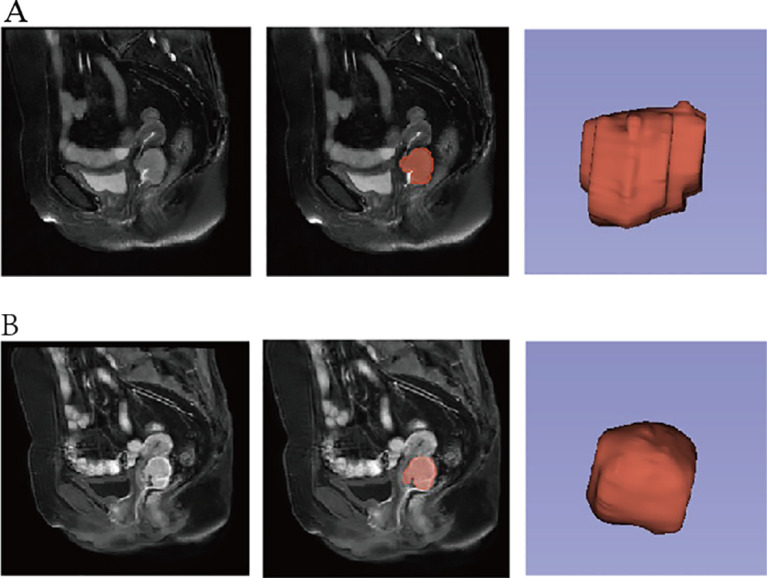
Manual outlining of ROI: **(A)** sagittal T2WI image and **(B)** sagittal CE-T1WI image. ROI, region of interest; T2WI, T2-weighted imaging; CE-T1WI, contrast-enhanced T1-weighted imaging.

MR image data in DICOM format were collected for sagittal T2WI and contrast-enhanced T1-weighted imaging (CE-T1WI) sequences. N4 bias field correction was performed on the images before image segmentation. Region of interest (ROI) outline on sagittal T2WI and CE-T1WI images was performed by an oncology radiotherapist by first manually outlining using the 3D Slicer software (version 4.11.0) (https://www.slicer.org/). ROI outline along the tumor margins was performed to cover the entire tumor including areas of intra-tumor hemorrhage, cystic degeneration, or necrosis. The ROIs were manually sketched layer by layer to form a three-dimensional (3D) volume of interest (VOI) (**Figure 2**). The ROIs were checked by an experienced oncology radiotherapist after all the ROIs were sketched.

### Radiomics feature extraction and screening

First, 99 sagittal T2WI and CE-T1WI images of cervical cancer cases were resampled to a voxel size of 1 * 1 * 1 mm^3^ to standardize voxel spacing. Voxel intensity values were discretized using a fixed bin width of 25 SI. The images were also normalized to reduce the differences in signal intensity of images captured by different machines. Then, the ROIs of all cases were subjected to radiomics feature extraction by the 3D Slicer software using the “Pyratomics” package of open-source Python software (https://pypi.org/project/pyradiomics/). ROI outlining and feature extraction were performed again for 30 randomly selected cases, and the intraclass correlation coefficient (ICC) was calculated to select features with an ICC > 0.75. The data were then normalized to 0 to 1, and features with *p* < 0.05 were screened using the t-test or U-test. Pearson’s correlation coefficient was then calculated by correlation analysis to identify redundant features, and features with correlation coefficients ≥ 0.9 were removed. Finally, redundancy features were further removed using the least absolute shrinkage and selection operator (LASSO) algorithm.

### Construction of the model

Eleven machine learning algorithms were used to develop radiomics models. Clinical features with statistically significant differences in the training group (*p* < 0.05) were used to construct a clinical prediction model based on the k-nearest neighbor (KNN) machine learning algorithm. A logistic regression algorithm was applied to construct a preoperative prediction model for the risk status of cervical cancer recurrence by combining clinical models with radiomics models and visualizing in nomograms. The nomograms generated individual probabilities of clinical events by integrating different prognostic and deterministic variables, thus meeting the needs of clinical practitioners for integrated biological and clinical models (13).

### Evaluation and validation of models

The predictive performance of the risk model was quantified by the AUC value of the area under the receiver operating characteristic (ROC) curve and the corresponding 95% confidence interval. It is generally considered that an AUC < 0.6 has poor discriminatory ability, an AUC of 0.6–0.75 has some discriminatory ability, and an AUC > 0.75 has good discriminatory ability (14). Other indicators for evaluating the model in the training and validation groups were calculated separately, including accuracy, sensitivity, and specificity. Decision curve analysis (DCA) was applied to assess the clinical utility value of the model.

### Statistical analysis methods

The statistics for this study were analyzed using the R software (version 4.2.2), and all statistical tests were two-sided, with *p* < 0.05 considered a statistically significant difference. Continuous variables were analyzed using a t-test or Wilcoxon rank sum test, and categorical variables were analyzed using the chi-square test.

### Ethics approval and consent to participate

Studies involving human participants were reviewed, processed, and approved, and data analysis was conducted in accordance with the Declaration of Helsinki. All experiments involving human participants were approved by the Ethics Committee of the Affiliated Hospital of North Sichuan Medical College (No. 2023ER327-1). As this was a retrospective case–control study and the cases were obtained from an electronic case bank, exemption from informed consent was granted by the Ethics Committee.

## Results

### Characteristics of the patients

A total of 99 cases of patients with operable cervical cancer were included and randomly divided into a training group (n = 79) and a testing group (n = 20). General clinical characteristics, age, number of pregnancies, number of deliveries, number of miscarriages, FIGO stage, menstrual status, and blood indices FAR, LMR, NLR, PLR, SIRI, PNI, SII, RDW, Hb, and SCCA were not statistically different between the training and test groups (*p* > 0.05) (**Table 1**). There was a statistical difference in PLR, SCCA, and number of deliveries between the PR and LR groups in the training group (*p* < 0.05) (**Table 2**).

**Table 1:** Clinical characteristics between the training group and the testing group.

Clinical characteristics	Training group (n = 79)	Testing group (n = 20)	*p*
Age	51.94 ± 10.18	52.90 ± 8.96	0.312
Number of pregnancies	4.00 [3.00, 5.50]	4.00 [3.00, 5.00]	0.518
Number of deliveries	2.00 [2.00, 2.50]	2.00 [2.00, 2.00]	0.55
Number of abortions	2.00 [1.00, 3.00]	2.00 [1.00, 2.25]	0.793
FAR	0.07 [0.07, 0.09]	0.07 [0.07, 0.09]	0.828
LMR	4.68 [3.86, 5.98]	4.48 [3.81, 5.26]	0.236
NLR	2.69 [1.73, 3.40]	2.83 [2.03, 3.41]	0.632
PLR	143.27 [116.72, 179.38]	141.15 [111.64, 210.16]	0.741
SIRI	0.77 [0.50, 1.09]	1.01 [0.73, 1.28]	0.115
PNI	51.74 ± 4.54	52.90 ± 4.55	0.312
SII	540.74 [394.11, 798.70]	677.00 [482.89, 765.00]	0.347
RDW	13.00 [12.70, 13.60]	13.25 [12.70, 13.75]	0.309
Hb	44.20 [42.40, 46.35]	45.30 [41.88, 45.99]	0.625
SCCA			0.496
<2 ng/mL	53 (67.09%)	15 (75.00%)	
≥2 ng/mL	26 (32.91%)	5 (25.00%)	
Menopausal state			0.887
Non-menopausal	29 (36.71%)	7 (35.00%)	
Menopausal	50 (63.29%)	13 (65.00%)	

FAR, albumin-to-fibrinogen ratio; LMR, lymphocyte-to-monocyte ratio; NLR, neutrophil-to-lymphocyte ratio; PLR, platelet-to-lymphocyte ratio; SIRI, systemic inflammatory response index; PNI, prognostic nutritional index; SII, systemic immunoinflammatory index; RDW, red cell distribution width; Hb, hemoglobin; SCCA, squamous cell carcinoma antigen.

**Table 2:** Clinical characteristics between the PR and the LR in the training group.

Clinical characteristics	LR group	PR group	*p*
Age	51.13 ± 10.45	52.73 ± 9.97	0.489
Number of pregnancies	4.00 [2.00, 5.00]	4.00 [3.00, 6.00]	0.186
Number of deliveries	2.00 [1.00, 2.00]	2.00 [2.00, 3.00]	0.033
Number of abortions	2.00 [1.00, 3.00]	2.00 [1.00, 3.00]	0.944
FAR	0.07 [0.07, 0.09]	0.07 [0.07, 0.08]	0.433
LMR	4.64 [4.18, 5.98]	4.74 [3.71, 5.94]	0.641
NLR	2.69 [1.73, 3.06]	2.67 [1.77, 3.67]	0.424
PLR	134.48 [112.83, 166.19]	153.12 [125.15, 202.17]	0.045
SIRI	0.73 [0.54, 0.98]	0.79 [0.49, 1.15]	0.543
PNI	51.00 ± 4.87	52.47 ± 4.12	0.149
SII	555.31 [359.05, 764.76]	510.86 [430.45, 881.19]	0.468
RDW	13.00 [12.60, 13.30]	13.05 [12.80, 13.72]	0.202
Hb	128.00 [117.00, 133.00]	128.00 [118.50, 134.00]	0.914
SCC			<0.001
<2 ng/mL	18 (46.15%)	35 (87.50%)	
≥2 ng/mL	21 (53.85%)	5 (12.50%)	
Menopausal state			0.75
non-menopausal	15 (38.46%)	14 (35.00%)	
menopausal	24 (61.54%)	26 (65.00%)	

LR, low risk; PR, positive risk; FAR, albumin-to-fibrinogen ratio; LMR, lymphocyte-to-monocyte ratio; NLR, neutrophil-to-lymphocyte ratio; PLR, platelet-to-lymphocyte ratio; SIRI, systemic inflammatory response index; PNI, prognostic nutritional index; SII, systemic immunoinflammatory index; RDW, red cell distribution width; Hb, hemoglobin; SCC, squamous cell carcinoma antigen.

The results of pathological features of all cases were compared (**Table 3**). There were 39 and 10 cases in the PR group and 40 and 10 cases in the LR group in the training and test groups, respectively. There were 67 and 12 cases of squamous and non-squamous carcinoma in the training group and 15 and 5 cases of squamous and non-squamous carcinoma in the testing group, respectively. Between the training and test groups, lymph node metastasis (negative/positive), paravaginal infiltration (negative/positive), vaginal margin (negative/positive), LVSI (negative/positive), DSI (>1/3 or ≤1/3), TS (≥4 cm or <4 cm), histological type (squamous/non-squamous), and risk stratification differences were not statistically significant (*p* > 0.05).

**Table 3:** Comparison of pathological characteristics in the training and test groups.

Pathological characteristics	Training group	Testing group	*p*
Lymph No. (%)			
Negative	15 (18.99%)	3 (15.00%)	0.929
Positive	64 (81.01%)	17 (85.00%)	
Paravaginal infiltration No. (%)			
Negative	4 (5.06%)	1 (5.00%)	1
Positive	75 (94.94%)	19 (95.00%)	
Vaginal margin No. (%)			
Negative	4 (5.06%)	0 (0.00%)	0.58
Positive	75 (94.94%)	20 (100.00%)	
TS No. (%)			
<4 cm	65 (82.28%)	15 (75.00%)	0.46
≥4 cm	14 (17.72%)	5 (25.00%)	
DSI No. (%)			
≤1/3	47 (59.49%)	14 (70.00%)	0.388
>1/3	32 (40.51%)	6 (30.00%)	
LVSI No. (%)			
Negative	24 (30.38%)	7 (35.00%)	0.691
Positive	55 (69.62%)	13 (65.00%)	
Histological type			
Squamous	67 (84.81%)	15 (75.00%)	0.326
Non-squamous	12 (15.19%)	5 (25.00%)	
Risk stratification			
Low	40 (50.63%)	10 (50.00%)	0.939
Medium	18 (22.78%)	4 (20.00%)	
High	21 (26.58%)	6 (30.00%)	
FIGO staging			0.740
Stage IA	6 (7.59%)	1 (5.00%)	
Stage IB	48 (60.76%)	14 (70.00%)	
Stage IIA	25 (31.65%)	5 (25.00%)	

TS, tumor size; DSI, depth of interstitial infiltration; LVSI, lymphovascular interstitial infiltration; FIGO, International Federation of Gynecology and Obstetrics.

### Radiomics features

**Figure 3:**
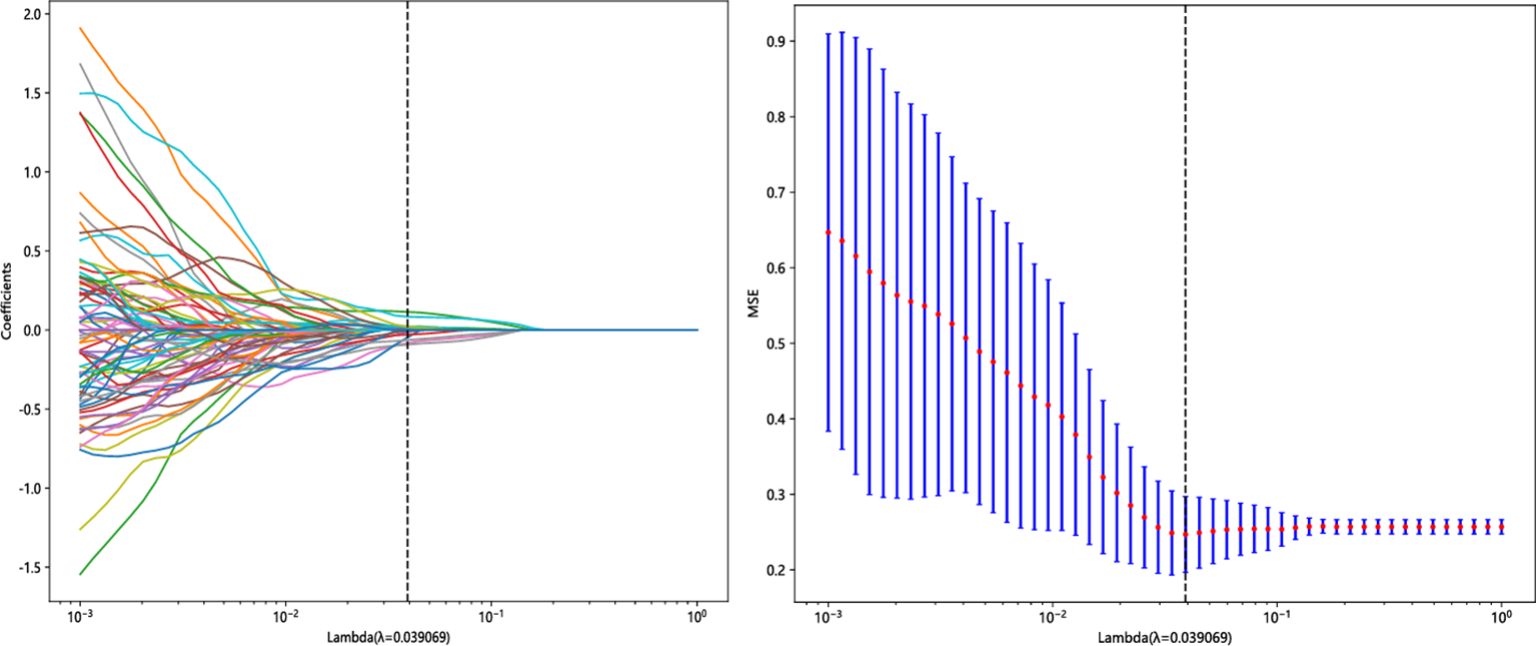
Radiomics feature screening using the LASSO algorithm. LASSO, least absolute shrinkage and selection operator.

**Figure 4:**
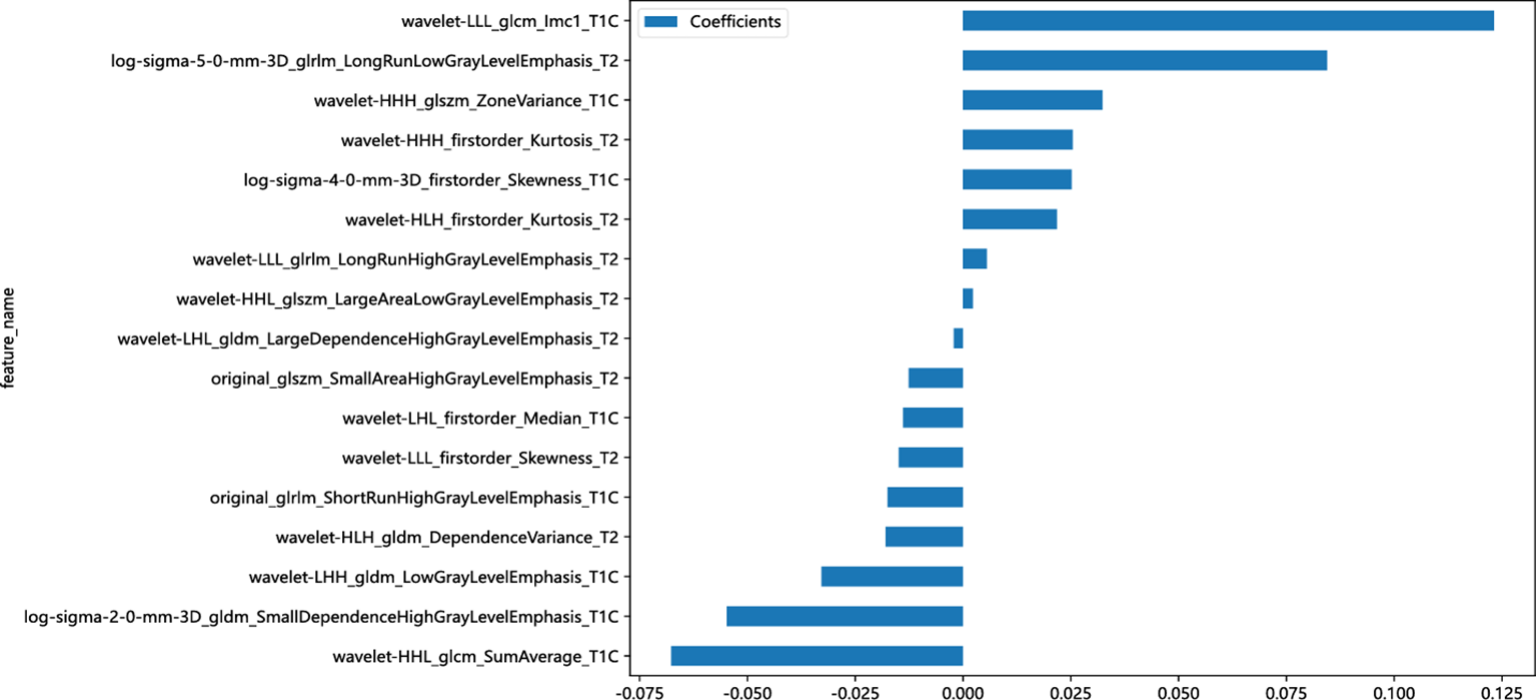
Weighted bar chart of key radiomics features.

A total of 1,223 radiomics features were extracted from the images of the T2WI and CE-T1WI sequences of the training group, respectively. A total of 2,446 features were extracted from the two sequences, including 468 first-order features (firstorder), 624 texture features (glcm), 364 texture features (gldm), 416 texture features (glrlm), 416 texture features (glszm), 112 texture features (ngtdm), and 28 morphological features (shape). Among all the extracted features, 1,821 features were retained by screening the repeatable features by ICC > 0.75. Then, features with *p* < 0.05 were retained by t-test or U-test, totaling 575. Then, using Pearson’s correlation analysis, features with correlation coefficients >0.9 were excluded, and a total of 121 features were retained for both sequences. Finally, 17 key radiomics features were filtered out after dimensionality reduction by the LASSO algorithm (**Figure 3**). The weights of the 17 radiomics features (**Figure 4**) and the Rad-score formula were calculated.

Rad-score Formula: 0.482845896481464 − 0.017516 * original_glrlm_ShortRunHighGrayLevelEmphasis_T1C − 0.054809 * log-sigma-2-0-mm-3D_gldm_SmallDependenceHighGrayLevelEmphasis_T1C + 0.025203 * log-sigma-4-0-mm-3D_firstorder_Skewness_T1C − 0.013897 * wavelet-LHL_firstorder_Median_T1C − 0.032844 * wavelet-LHH_gldm_LowGrayLevelEmphasis_T1C − 0.067680 * wavelet-HHL_glcm_SumAverage_T1C + 0.032331 * wavelet-HHH_glszm_ZoneVariance_T1C + 0.123124 * wavelet-LLL_glcm_Imc1_T1C − 0.012611 * original_glszm_SmallAreaHighGrayLevelEmphasis_T2 + 0.084444 * log-sigma-5-0-mm-3D_glrlm_LongRunLowGrayLevelEmphasis_T2 − 0.002119 * wavelet-LHL_gldm_LargeDependenceHighGrayLevelEmphasis_T2 + 0.021768 * wavelet-HLH_firstorder_Kurtosis_T2 − 0.017925 * wavelet-HLH_gldm_DependenceVariance_T2 + 0.002260 * wavelet-HHL_glszm_LargeAreaLowGrayLevelEmphasis_T2 + 0.025433 * wavelet-HHH_firstorder_Kurtosis_T2 − 0.014920 * wavelet-LLL_firstorder_Skewness_T2 + 0.005486 * wavelet-LLL_glrlm_LongRunHighGrayLevelEmphasis_T2.

### Model construction and validation

**Figure 5:**
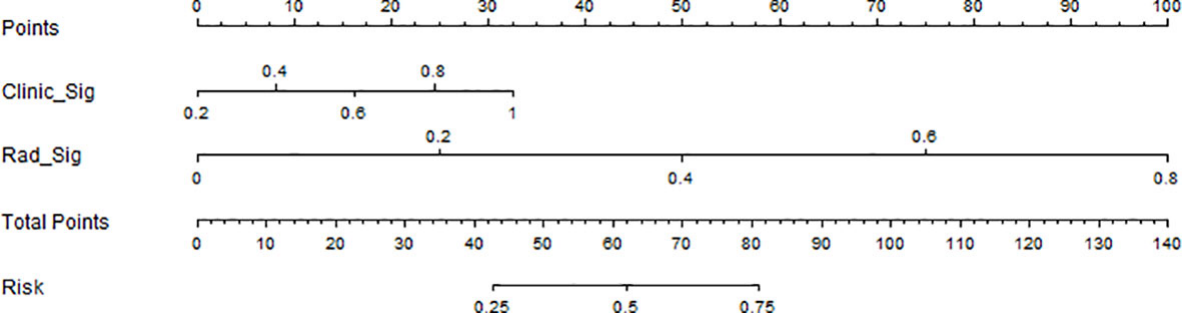
Nomogram of fused clinical-radiomics models.

Radiomics models were constructed by 11 machine learning algorithms, and the diagnostic performance of different radiomics models is shown in **Table 4**. Combining the AUC values, accuracy, sensitivity, and specificity of each model in the training and test groups, KNN was considered the best machine learning algorithm for radiomics model building. Clinical features with significant differences in the training group included PLR, SCCA, and number of deliveries, and the clinical prediction model (Clinic_Sig) was constructed based on the KNN algorithm. The fusion model of the clinical model combined with the radiomics model was constructed by logistic regression algorithm and visualized in the nomogram (**Figure 5**).

**Table 4:** Predictive efficacy of radiomics models with 12 machine learning algorithms.

Cohort	Model	Accuracy	AUC	95% CI	Sensitivity	Specificity	Precision
Training	LR	0.785	0.882	0.808–0.956	0.795	0.775	0.775
Testing	LR	0.750	0.840	0.645–1.000	0.700	0.800	0.778
Training	NaiveBayes	0.759	0.852	0.770–0.934	0.667	0.850	0.813
Testing	NaiveBayes	0.750	0.850	0.678–1.000	0.700	0.800	0.778
Training	SVM	0.937	0.962	0.925–0.999	0.897	0.975	0.972
Testing	SVM	0.800	0.810	0.606–1.000	0.700	0.900	0.875
Training	KNN	0.747	0.892	0.826–0.958	0.590	0.900	0.852
Testing	KNN	0.800	0.860	0.677–1.000	0.700	0.900	0.875
Training	RandomForest	0.962	0.995	0.987–1.000	0.974	0.950	0.950
Testing	RandomForest	0.750	0.840	0.637–1.000	0.700	0.800	0.778
Training	ExtraTrees	1.000	1.000	1.000	1.000	1.000	1.000
Testing	ExtraTrees	0.650	0.845	0.666–1.000	0.600	0.700	0.667
Training	XGBoost	1.000	1.000	1.000	1.000	1.000	1.000
Testing	XGBoost	0.700	0.820	0.616–1.000	0.700	0.700	0.700
Training	LightGBM	0.823	0.882	0.809–0.956	0.795	0.850	0.838
Testing	LightGBM	0.450	0.635	0.353–0.917	0.300	0.600	0.429
Training	GradientBoosting	0.962	0.992	0.977–1.000	0.949	0.975	0.974
Testing	GradientBoosting	0.700	0.725	0.475–0.975	0.700	0.700	0.700
Training	AdaBoost	0.911	0.982	0.962–1.000	0.949	0.875	0.881
Testing	AdaBoost	0.700	0.855	0.688–1.000	0.800	0.600	0.667
Training	MLP	0.835	0.929	0.875–0.983	0.795	0.875	0.861
Testing	MLP	0.850	0.850	0.666–1.000	0.800	0.900	0.889

AUC, area under the curve; LR, logistic regression; SVM, support vector machine; KNN, k-nearest neighbor.

**Figure 6:**
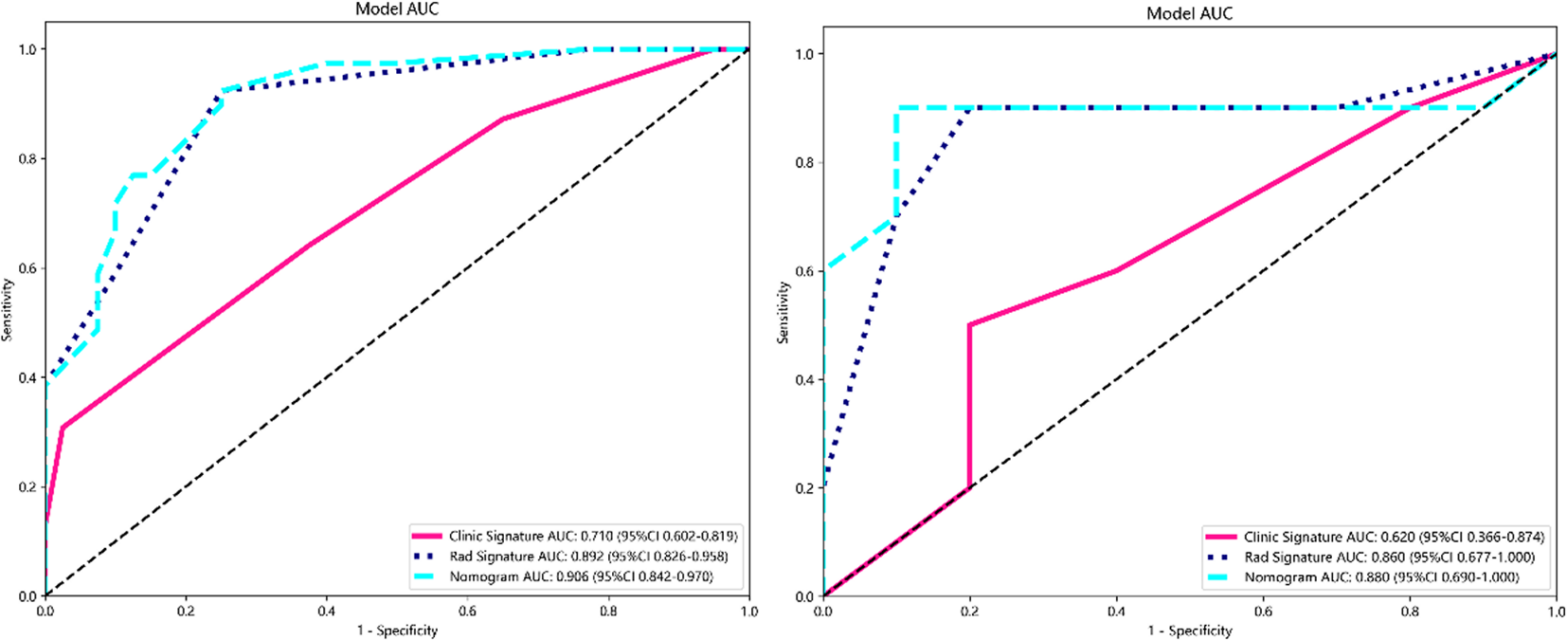
ROC curves for clinical, radiomics, and fusion models in the training group (left) and testing group (right). ROC, receiver operating characteristic.

**Figure 7:**
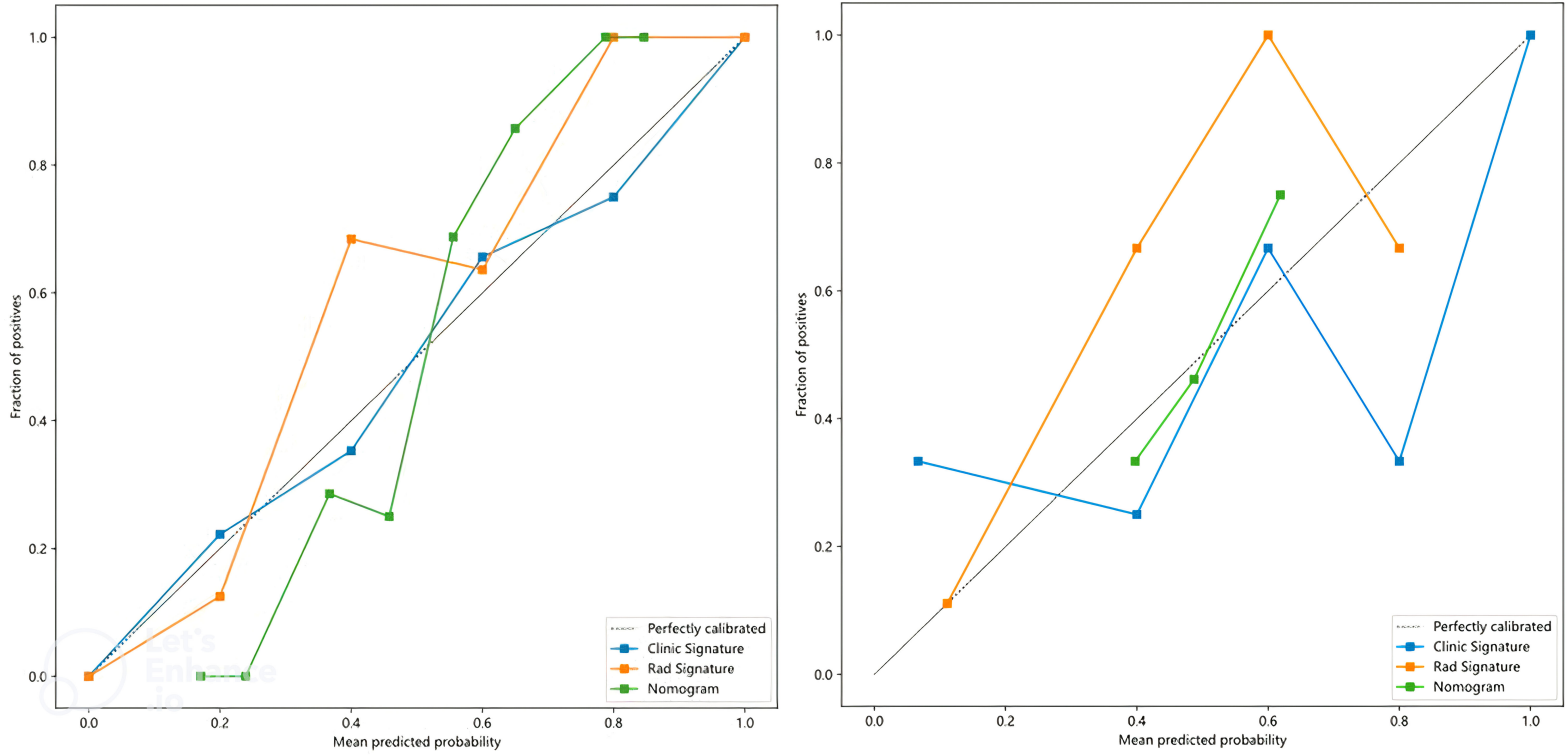
Calibration curves for clinical, radiomics, and fusion models in the training group (left) and testing group (right).

**Figure 8:**
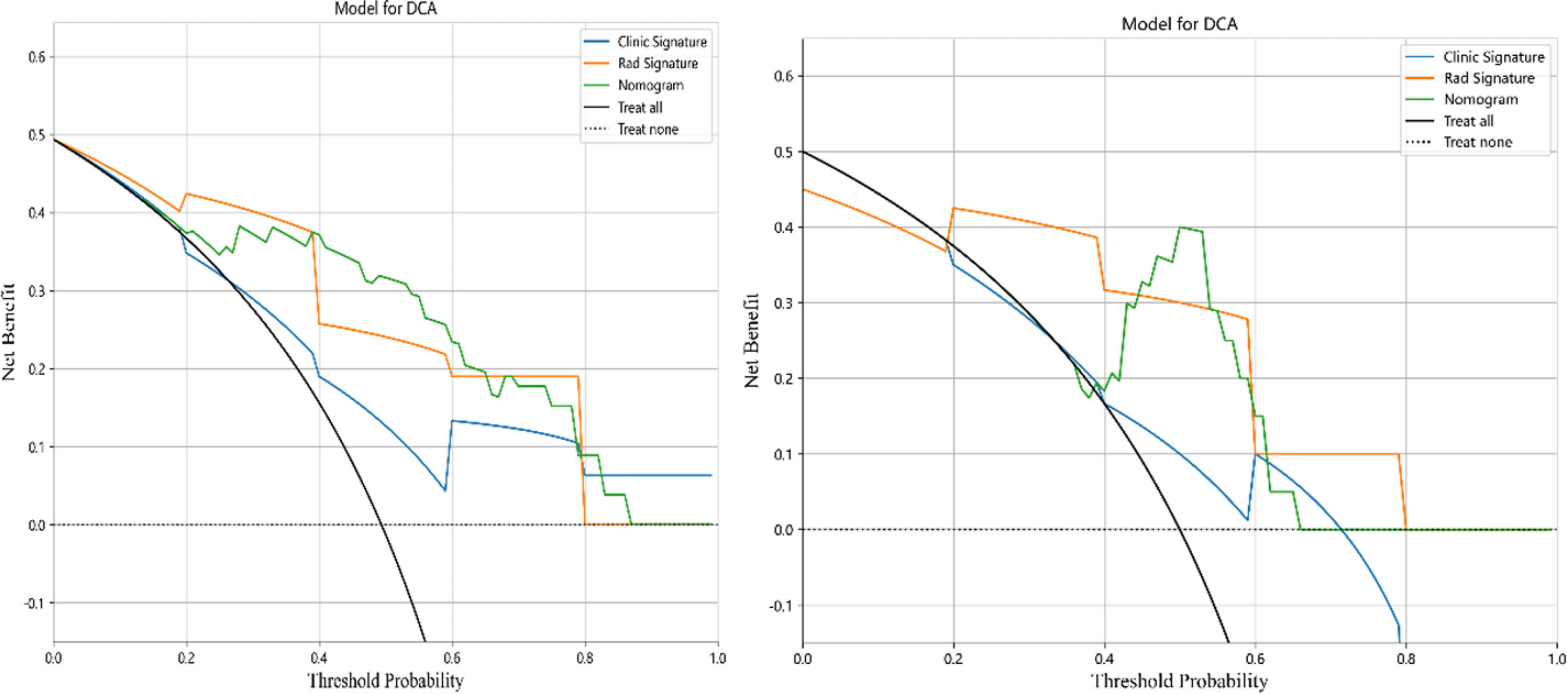
Decision curves for clinical, radiomics, and fusion models in the training group (left) and testing group (right).

In this study, the accuracy, AUC value, sensitivity, specificity, and precision of the three predictive models finally constructed were compared (**Table 5**). The AUC values of the clinical model, the radiomics model, and the fusion model in the training group were 0.710 (95% CI 0.602–0.819), 0.892 (95% CI 0.826–0.958), and 0.906 (95% CI 0.842–0.970), respectively, and the AUC values in the testing group were 0.620 (95% CI 0.366–0.874), 0.860 (95% CI 0.677–1.000), and 0.880 (95% CI 0.690–1.000), respectively (**Figure 6**). The fusion model had an accuracy of 82% and 90%, sensitivity of 77% and 90%, specificity of 88% and 90%, and precision of 86% and 90% in the training and test groups, respectively. The AUC values of the fusion model were higher than those of the clinical and radiomics models in both the training and test groups, and the fusion model had the best predictive efficacy. The DeLong test was used to compare the differences between the AUC values of the models, and the results showed that there was a statistically significant difference between the AUC values of the fusion model and the clinical model (*p* < 0.05), and the difference between the AUC values of the fusion model and the radiomics model was not statistically significant (*p* > 0.05). For the calibration curves of the three models in the training and testing groups (**Figure 7**), the *p*-values of the Hosmer–Lemeshow test were 0.589 and 0.101 in the training and testing groups of the fusion model, respectively, indicating that there is a better agreement in the predicted and actual values. The comparison of the decision curves of the three models for both the training and test groups showed that, in most cases, the fusion model brought more net gain in most of the threshold probability range and brought the greatest net gain when the threshold probability was approximately 0.5 (**Figure 8**).

**Table 5:** Predictive efficacy of different models in training and testing groups.

Cohort	Model	Accuracy	AUC	95% CI	Sensitivity	Specificity	Precision
Training	Clinic_Sig	0.633	0.710	0.602–0.819	0.641	0.625	0.625
Training	Rad_Sig	0.747	0.892	0.826–0.958	0.590	0.900	0.852
Training	Nomogram	0.823	0.906	0.842–0.970	0.769	0.875	0.857
Testing	Clinic_Sig	0.600	0.620	0.366–0.874	0.600	0.600	0.600
Testing	Rad_Sig	0.800	0.860	0.677–1.000	0.700	0.900	0.875
Testing	Nomogram	0.900	0.880	0.690–1.000	0.900	0.900	0.900

AUC, area under the curve.

## Discussion

In early-stage cervical cancer patients, radical surgical treatment or CCRT is feasible, both of which have comparable therapeutic efficacy (15), whereas postoperative patients receive postoperative adjuvant therapy based on pathological risk factors and stage. The prognosis of patients with cervical cancer is closely related to the presence of risk factors, and the 3-year recurrence rate of patients with intermediate-risk factors increased from 2% to 31% compared with those without intermediate-risk risk factors, and postoperative adjuvant therapy can significantly reduce the recurrence rate and mortality (16). High-risk factors increase the postoperative recurrence rate of patients with early-stage cervical cancer by approximately 40%, and the 5-year recurrence-free survival rate decreases from 80%–90% to 40%–70% (17). Clinicopathological evaluation should be performed before radical surgical resection in patients with cervical cancer, which is beneficial for treatment planning and may avoid toxic side effects from multimodality therapy (18). In this study, an individualized prediction model for cervical cancer recurrence risk stratification was constructed based on preoperative bp-MRI images combined with clinical data from patients with operable cervical cancer, and the results showed that the fusion model had the best predictive efficacy, with AUC values of 0.906 (95% CI 0.842–0.970) for the training group and 0.880 (95% CI 0.690–1.000) for the testing group. This enables preoperative risk stratification of cervical cancer patients, which is useful in supporting the clinical development of individualized treatment decisions.

In this study, ROI outlining covered the entire tumor, including areas of intra-tumor hemorrhage, cystic degeneration, or necrosis, in order to reduce the impact of intra-tumor heterogeneity on radiomics features. Radiomics has been widely applied to a variety of tumors, demonstrating that quantitative imaging histological features are associated with tumor pathological characteristics, survival outcomes, identification of lymph node metastases, and assessment of tumor response to treatment, facilitating improved decision support in a cost-effective and non-invasive manner. More hematological indexes including LMR, NLR, PLR, SIRI, PNI, SII, RDW, Hb, FAR, and SCCA were incorporated into the clinical baseline characteristics of this study. The analysis of the difference between the PR group and the LR group in the training group showed that there was a statistically significant difference in the number of deliveries of PLR and SCCA. A clinical prediction model based on the KNN algorithm was constructed with AUC values of 0.765 and 0.620 for the training group and 0.765 and 0.620 for the testing group, and their predictive efficacy was significantly inferior to that of the radiomics model and the fusion model. In this study, a maximum tumor diameter of 4 cm was used as the threshold value for assessment in risk stratification of cervical cancer. However, it has been reported that a maximum tumor diameter greater than 2 cm is a prognostic risk factor for cervical cancer (19), and the FIGO staging of cervical cancer (2018 version) uses a maximum tumor diameter line of 2 cm as a threshold value for stage (20). Therefore, future studies are necessary to further explore the impact of different criteria on risk stratification. In this study, the AUC values of the radiomics model with combined T2WI and CE-T1WI sequences were 0.893 and 0.825 in the training and test groups, respectively, and the AUC values of the clinical-radiomics fusion model with combined clinical predictive parameters were 0.913 and 0.850 in the training and test groups, respectively; the AUC values of the fusion model were significantly higher, and the predictive efficacy was better. The DeLong test showed no statistically significant difference in the AUC values between the fusion model and the radiomics model (*p* > 0.05). This may be due to the fact that the radiomics model alone can show better predictive efficacy, whereas the limited number of case samples in this study, as well as the fewer features used to build the clinical model, necessitates future analysis of large data samples.

Postoperative risk factors for recurrence of cervical cancer include pelvic lymph node metastasis, surgical margins and paracervical infiltration, tumor diameter, deep mesenchymal infiltration, lymphovascular infiltration, and other independent prognostic factors, stratified according to different risk classes (21). Cervical cancer patients presenting an intermediate risk have up to a 30% recurrence and mortality risk rate after surgery alone (22), and high-risk patients not receiving adjuvant therapy have a 40% recurrence rate and a 50% mortality risk rate (23). Du W et al. (9) predicted lymphovascular infiltration in cervical cancer based on preoperative MR images, constructed a radiomics model with a clinical model with AUC values of 0.925 and 0.786 in the training group and 0.911 and 0.706 in the testing group, and produced the best diagnostic performance in the fusion model of the combined radiomics features with the clinical data, with an AUC of 0.943 in the training group and an AUC of 0.923 in the testing group. Benedetti-Panici et al. (10) showed that subclinical paracervical spread occurs in approximately 30%–60% of early-stage cervical cancers and that pelvic lymph node metastasis is always associated with paracervical infiltration. In early-stage cervical cancer, 37% of parauterine invasion was through direct invasion, 59% through lymph node metastasis, and 52% of cases through LVSI. For patients with locally advanced cervical cancer with a risk of recurrence, neoadjuvant chemotherapy followed by surgery cannot be considered a quasi-treatment (24), and the identification of risk factors prior to treatment is important for the development of the treatment plan. Furthermore, it was found that lymphatic metastasis in cervical cancer does not exist as a biological behavior alone and is subject to the imaging of a variety of clinicopathological features including the degree of differentiation of the tumor, the depth of interstitial infiltration, and the paraventricular infiltration (25). The outcomes of radical surgery and radiotherapy are comparable for patients with early-stage cervical cancer, but the combination of surgery and adjuvant radiotherapy increases the incidence of treatment-related adverse events. For example, patients receiving adjuvant therapy after radical hysterectomy have a higher incidence of urinary complications, such as ureteral stenosis, and vascular and lymphatic complications, such as lower limb edema. Therefore, it is recommended to accurately assess the risk status of recurrence of in operable cervical cancer preoperatively and to convert patients who may require adjuvant therapy after surgery to radiotherapy (26). In this study, three prediction models for preoperative prediction of the recurrence risk status of operable cervical cancer were constructed by machine learning methods, and it was found that the fusion model had the best prediction performance, which can be used to assist the clinic to more accurately stratify patients’ recurrence risk preoperatively, which can help to further guide the individualized treatment.

### Limitations and future research directions

Although this study made some important findings in predicting the risk of recurrence of early-stage cervical cancer, there are still some limitations. First, this study lacked an independent testing group. An independent testing set should be completely isolated from the feature selection, model selection, and model construction processes to more reliably assess the generalization ability of the model. The lack of an independent testing group may result in a model that performs well on training data but has insufficient predictive power on new data. In addition, the construction and validation of the model in this study were based on single-center, small-sample data, and the results may be overfitting; multicenter, large-sample data are needed to validate and optimize the model in the future to improve the clinical generalizability of the model. Future studies should expand the sample size to include multicenter data and use rigorous external validation methods to ensure model robustness and applicability. Nonetheless, this study provides valuable exploratory data and preliminary evidence that provides direction for further research. Researchers should continue to explore and validate predictive models for the risk of recurrence in early-stage cervical cancer, with the aim of providing a more reliable and effective tool in clinical practice.

Although radiomics has shown great potential for prognostic prediction in cervical cancer, it still faces some implementation challenges in practical clinical applications. The performance of radiomics models relies heavily on the quality and consistency of imaging data. However, imaging data from different healthcare organizations and different devices often differ, which may lead to compromised predictive performance of the models. Standardization and harmonization of imaging data remain a pressing issue today. Data sharing across organizations is often challenging due to privacy protection and data security issues. Validation studies involving multiple centers are essential in order to enhance the generalization ability of models. However, sharing data across institutions requires overcoming multiple legal, ethical, and technological barriers. The widespread use of radiomics technology requires that clinical staff have a technical background in understanding and interpreting model results, which may require additional training and education. Therefore, the acceptance of this emerging technology by physicians and radiologists and how effectively it can be integrated into the clinical decision-making process are factors that must be considered in future implementations. With the continuous development and improvement of the technology, it is expected that these barriers will be gradually resolved to provide more solid support for the precision treatment of cervical cancer.

## Conclusion

In this study, an individualized prediction model stratified by risk factors for recurrence of operable cervical cancer was constructed based on the radiomics features of preoperative bp-MRI images, and the clinical–radiomics fusion model showed the best efficacy in terms of predictive value. MRI radiomics presents an important contribution to the preoperative prediction of early-stage cervical cancer recurrence risk status that can be used preoperatively and is expected to provide a more intuitive aid to clinical decision-making. However, as this study is a single-center study with a small sample size, the lack of an independent testing set and multicenter data may affect the generalizability and stability of the results. Therefore, the findings of this study should be considered preliminary exploratory results, and further large-scale, multicenter studies are needed to validate and extend these findings.

## Data Availability

The original contributions presented in the study are included in the article/supplementary material. Further inquiries can be directed to the corresponding authors.
